# Nature in Disease; and Other Writings

**Published:** 1855-01

**Authors:** 


ART. IX.—*Nature in Disease; and other writings.* By JACOB BIGELOW, M. D., Prof. of Clinical Medicine and Materia Medica in the Massachusetts Medical College. Boston: Ticknor & Fields. 1854.

This is a little duodecimo made up of a number of brief essays on medical topics, some of them practical and others being introductory lectures before medical classes, or addresses before learned societies.

All of these are reliable, and some of them extremely interesting. Dr. Bigelow is without parade as a writer—his style, though sufficiently illustrated, is still severe, precise and neat; got up upon the Boston model of Puritanic simplicity.

Those who read this little volume will find in it a large amount of common sense and straightforward argument. At the same time we must enter our protest against the doctrines of his report to the Council of the Mass. Medical Society upon the toleration of Homœpaths within the membership. The old argument, that the true way to kill quackery is by giving a silent assent to its respectability, is used very ingeniously by Dr. Bigelow; but we think that the only question for that old and respectable society should have been, first, is Homœopathy quackery? and second, shall we place it upon a level with ourselves?

For sale by MILLER, ORTON & MULLIGAN. H.

